# The Effect of Repetitive Transcranial Magnetic Stimulation on Lower-Limb Motor Ability in Stroke Patients: A Systematic Review

**DOI:** 10.3389/fnhum.2021.620573

**Published:** 2021-09-01

**Authors:** Huiliu Fan, Yang Song, Xuanzhen Cen, Peimin Yu, István Bíró, Yaodong Gu

**Affiliations:** ^1^Faculty of Sports Science, Ningbo University, Ningbo, China; ^2^Doctoral School of Safety and Security Sciences, Obuda University, Budapest, Hungary; ^3^Faculty of Engineering, University of Szeged, Szeged, Hungary

**Keywords:** stroke, systematic review, walking, balance, repetitive transcranial magnetic stimulation

## Abstract

Repetitive transcranial magnetic stimulation (rTMS) is fundamental in inducing neuroplastic changes and promoting brain function restoration. Nevertheless, evidence based on the systematic assessment of the implication of rTMS in stroke patients is inadequate. This study aimed to evaluate the value of rTMS in the treatment of lower-limb motor dysfunction in stroke patients via gait characteristics. The electronic literature search was performed in ScienceDirect, Google Scholar, and PubMed databases using “repetitive transcranial magnetic stimulation,” “gait,” and “stroke” between 2000 and 2020. By screening all the identified studies, a total of 10 studies covering 257 stroke patients were included by matching the inclusion criteria, involving both rTMS with high (≥5 Hz) and low frequency (<5 Hz). Despite the limited study number and relatively high risk of bias, the results of this review primarily confirmed the enhancing effects of rTMS on the lower-limb motor ability (e.g., gait and balance) of stroke patients. In addition, 15- to 20-min course of rTMS for 2 to 3 weeks was found to be the most common setting, and 1 Hz and 10 Hz were the most commonly used low and high frequencies, respectively. These results might have significant clinical applications for patients with weakened lower-limb mobility after a stroke. Nevertheless, more rigorous studies in this field are much warranted.

**Systematic Review Registration:**https://inplasy.com/, identifier INPLASY202180079.

## Introduction

Stroke is an acute syndrome of clinical signs of focal (or global) disturbance of cerebral function, which can even lead to death (Wolfe, 2000). Disability is a common complication for patients who have survived a stroke. There are also some secondary changes in the skeletal muscles of stroke patients, including the reduced muscle mass as well as increased intramuscular fat (Scherbakov et al., 2013), which may further reduce the muscle strength and gait independence (Akazawa et al., 2017; Naoki et al., 2018). Eventually, these results would seriously influence the life quality of these patients (Wade et al., 1992). According to previous studies, nearly 50% of stroke patients would suffer from hemiparesis as well as 30% of them are unable to walk without assistance (Kelly-Hayes et al., 2003). Previous studies have also found that stroke patients walked slower compared to healthy individuals, and it can also be distinguished by the higher falling cadence, prolonged gait cycle, temporal gait asymmetry, and also increased double support phase after stroke (Von Schroeder et al., 1995; Patterson et al., 2008).

It was indicated that gait performance is an important index to evaluate lower extremity motor function recovery post-stroke (Barroso et al., 2017). Most stroke patients would obtain a certain degree of walking ability improvement after normal rehabilitation treatment, but most of them are accompanied by the abduction and external rotation of the hip joint, hyperextension of the knee joint, foot drop, varus, and short support time of the affected side, resulting in the gait abnormity (Byun et al., 2011). Evaluating the gait biomechanics of stroke patients has been applied to predict the improvement in functional ability after interventions (Kim and Eng, 2004). It also plays an important role in making rehabilitation strategies and monitoring its impact (Barroso et al., 2017). For example, the symmetry of the center of foot pressure in stroke patients is closely related to the degree of lower limb dysfunction, limb balance ability, stride speed, and body stability, which can reflect the degree of lower extremity function recovery and walking quality in stroke patients (Chen et al., 2007; Patterson et al., 2008).

Repetitive transcranial magnetic stimulation (rTMS), one of the brain stimulation techniques without any trauma, can be used to induce neuroplastic changes as well as promote brain function restoration (Clément et al., 2018). For the different brain functions, different intensities, frequencies, stimulation positions, and coil directions of rTMS need to be adjusted to achieve good therapeutic effects (Mansur et al., 2005; Machado et al., 2008). Previous research has proved that using rTMS can stimulate the representative brain areas of lower limb movement and further help patients improve motor function (Chieffo et al., 2014; Sasaki et al., 2016). For example, three weeks of high-frequency deep rTMS has a favorable influence on long-term improvements in the motor function of the lower extremity after stroke, and it could last for more than a month since the treatment finished (Chieffo et al., 2014). rTMS combined with traditional physiotherapy and occupational therapy can achieve a better rehabilitation effect on promoting motor function recovery (Cha and Kim, 2017). Besides, rTMS combined with repetitive transcranial electrical stimulation also contributed to the motor function recovery in stroke patients, which is more effective than combined limb function training (Attal et al., 2016). Tung et al. (2019) has already reviewed the short-term beneficial effects of rTMS on the post-stroke recovery of lower limb motor function, and the safety of applying rTMS has been further affirmed. Nevertheless, there is quite limited evidence based on the systematic assessment of the implication of rTMS in stroke patients up to now, let alone any conclusions regarding the better setting of rTMS interventions (e.g., frequency and duration) that could contribute to more benefit.

Therefore, the aim of this study was to primarily review and summarize the value of rTMS in the treatment of lower extremity motor dysfunction in stroke subjects via gait characteristics, and then try to find out the appropriate rTMS setting that may contribute to more benefits. The review could provide specific knowledge for researchers and clinicians on the use of rTMS in patients.

## Methods

### Search Strategy

The search strategy was applied to find out all the relevant published literature on the influence of rTMS on lower limb motor dysfunction in stroke patients. The extensive systematic search for all electronic publications from 2000 to 15 February 2020 was conducted using three databases including ScienceDirect, Google Scholar, and PubMed. The English-language literature search employed the following search words: “repetitive transcranial magnetic stimulation” AND “gait” AND “stroke”. The citation snowballing method was applied to identify other potentially relevant studies in the reference list of all eligible articles (Song et al., 2020). And these studies that have been accidentally overlooked were searched in other electronic databases to get available full-text by entering the specific information of authors and article titles.

In order to ensure a rigorous searching process, two researchers independently searched and assessed the retrieved literature. Any disagreements of inclusion (if existed) would be resolved with the third author.

### Eligibility Criteria

After deleting duplicate articles, the retrieved articles were screened first by titles then assessed by abstracts and full-text to meet the following eligibility criteria in accordance with the PICOS standard. (1) participants/patients, participants who have been diagnosed with stroke were included in this study, and there is no restriction on age, sex, and ethnicity; (2) Interventions, studies where participants received rTMS interventions or rTMS interventions combined with traditional physiotherapy and occupational therapy were included; (3) comparison, there is no limitation regarding the control interventions; (4) outcome, the main outcomes that collected from these included studies was the change of gait characteristics after interventions; (5) study design, English papers that published on peer-reviewed journals were covered, however, conference abstracts, review articles, book chapters, case reports, etc., were not included in this review.

### Data Extraction

For each published literature, the following information was extracted and summarized, and verified by two authors independently, characteristics of studies (i.e., authors and year), characteristics of subjects (i.e., sample size, age, gender, time post stroke, and treatment state), experimental design (i.e., coil type, intervention, control, and parameters), and primary outcomes. Mendeley software (Elsevier Ltd., Amsterdam, Netherlands) was applied to organize published literature and create citations. Any disagreements were handled by discussing or consulting with another author.

### Study Quality Assessment

According to the Cochrane Risk of Bias Assessment Tool, two researchers independently evaluated the quality of these included studies. More specifically, seven following aspects were assessed, (1) random sequence generation; (2) allocation concealment; (3) blinding of participants and personnel; (4) blinding of outcome assessment; (5) incomplete outcome data; (6) selective reporting; (7) other biases. Any disagreements were handled by discussing or consulting with another author.

## Results

### Search Results

**Figure 1:**
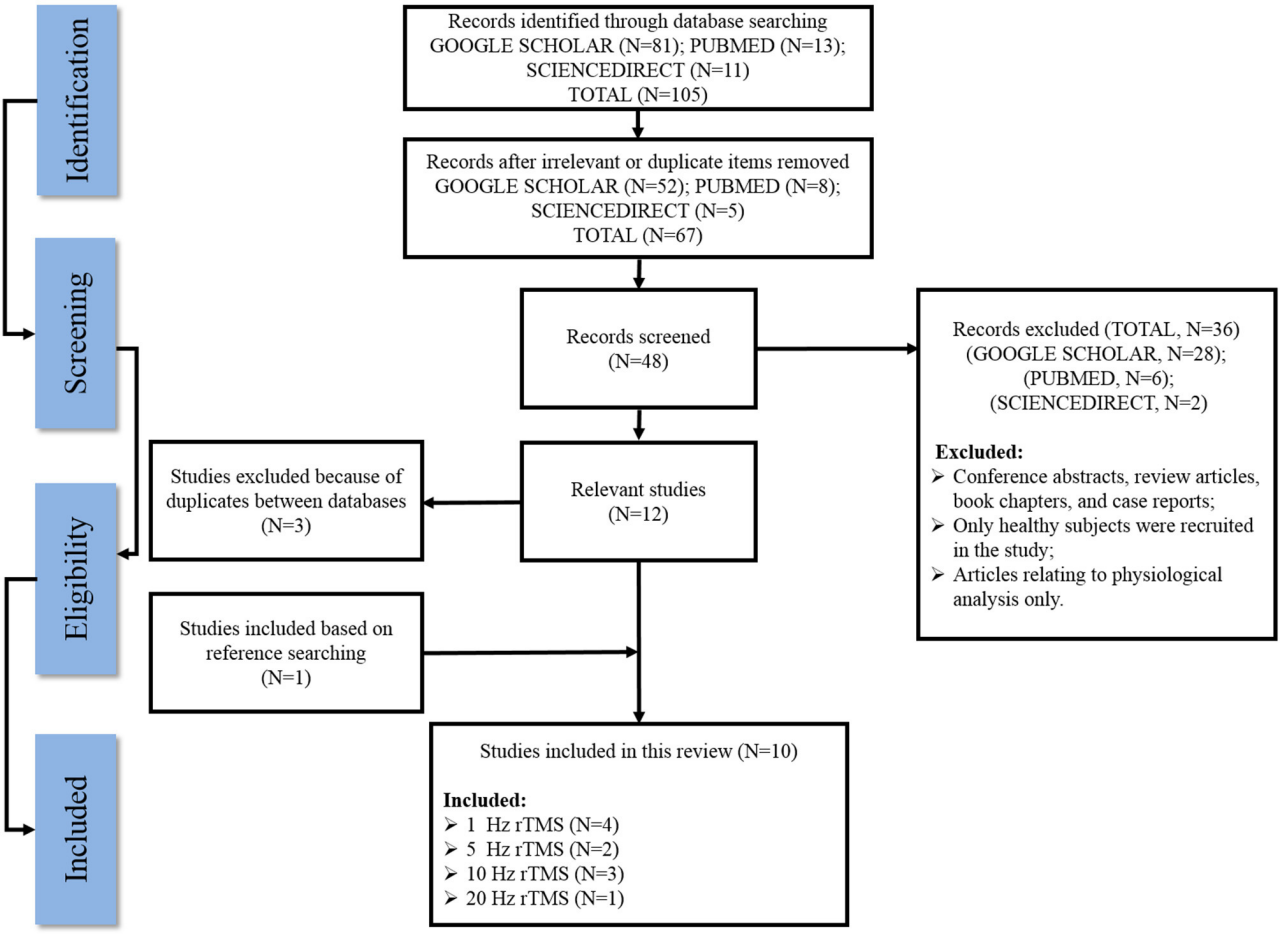
Flow diagram of the literature inclusion and exclusion process.

Figure 1 outlines a flow diagram illustrating the search history and selection protocol. The literature search produced a total of 105 records from three electronic databases (SCIENCEDIRECT, GOOGLE SCHOLAR, and PUBMED) and they were reduced to 67 after removing all the irrelevant/duplicate articles. Based on the eligibility criteria, 28 articles from GOOGLE SCHOLAR, six articles from PubMed, and two articles from SCIENCEDIRECT were excluded as potentially inappropriate for the current study. One additional article was included after checking the reference lists of the eligible articles, while three articles were removed owing to duplicates between these databases. Finally, a total of 10 articles that met the inclusion standard were included in the present systematic review.

### Study Quality

**Figure 2:**
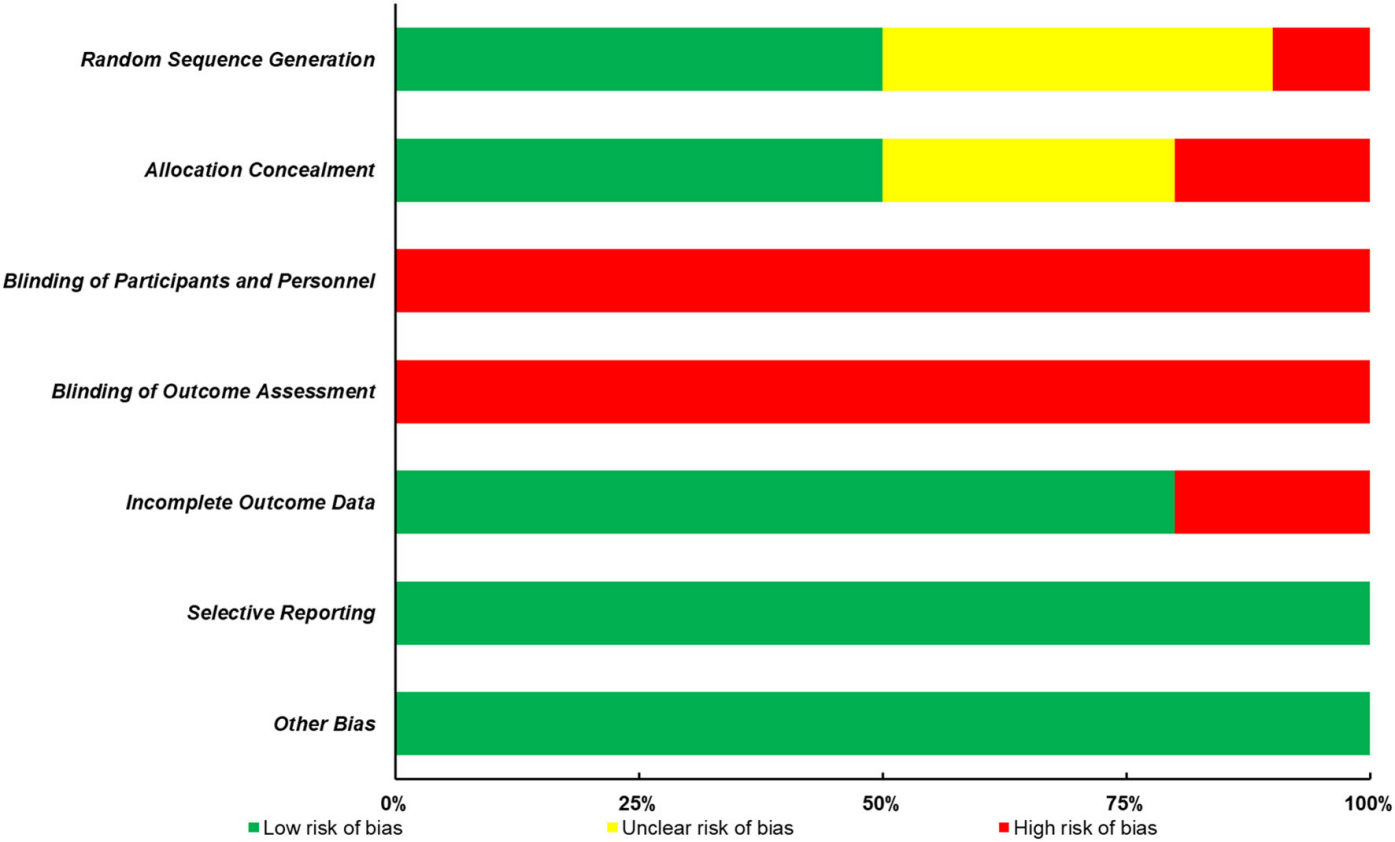
Risk of bias of the included studies.

The quality of all included studies was evaluated in terms of risk of bias, and the results were presented in Figure 2; Table 1. Firstly, it is always not possible to completely blind the participants and personnel to allocation and outcome. Thus, the following two aspects, blinding of participants and personnel and blinding of outcome assessment, contributed to the main sources of risk of bias. Furthermore, of all the 10 studies included in this review, only half of them presented the detailed methods of group randomization and used the allocation concealment, which further lowered the study quality. Nevertheless, a low risk of bias of the incomplete outcome data, selective reporting, and other biases domains was found in most studies.

**Table 1:** Risk of bias evaluation of included studies.

**Trials**	**Random Sequence Generation**	**Allocation Concealment**	**Blinding of Participants and Personnel**	**Blinding of Outcome Assessment**	**Incomplete Outcome Data**	**Selective Reporting**	**Other Bias**
Chieffo et al. (2014)	Unclear	Unclear	High	High	Low	Low	Low
Kim et al. (2014)	Unclear	High	High	High	Low	Low	Low
Rastgoo et al. (2016)	High	Unclear	High	High	Low	Low	Low
Choi et al. (2016)	Low	Low	High	High	Low	Low	Low
Wang et al. (2019)	Low	Low	High	High	High	Low	Low
Kakuda et al. (2013)	Low	High	High	High	High	Low	Low
Elkholy et al. (2014)	Low	Unclear	High	High	Low	Low	Low
Wang et al. (2012)	Unclear	Low	High	High	Low	Low	Low
Goh et al. (2020)	Unclear	Low	High	High	Low	Low	Low
Ji et al. (2016)	Low	Low	High	High	Low	Low	Low

### The Basic Characteristics of Included Studies

Tables 2, 3 summarized the basic characteristics of all the included studies. Of all the included studies published from 2000 to 2020, 257 stroke patients were covered in these studies, with the age ranged from less than 18 to 80 years old. The stimulus interventions can be classified as low-frequency (defined as <5 Hz) and high-frequency (defined as more than 5 Hz) based on the summary, with 1 Hz being the most commonly used low frequency and 10 Hz being the most commonly used high frequency. The duration of the intervention varied from 1 week to 6 weeks, with 2 to 3 weeks being the most common setting.

**Table 2:** The basic characteristics of the included studies.

**Trials**	**Sample size (N)**	**GenderM/F**	**Age (years) /time Post-Stroke (months)**	**Treatment State**	**Intervention**	**Coil type**	**Control**	**Experimental Design**	**Outcome Parameters**
**1. Low-frequency rTMS**									
Elkholy et al. (2014)	N: 30	16/14	rTMS: 44.06 ± 3.71/ 2.53 ± 0.52 CON: 45.66 ± 4.27/ 2.533 ± 0.516	Cerebrovascular accident	rTMS + physical therapy	Double-cone coil	Physical therapy	Frequency: 1 Hz Duration: 20 min x 3 sessions per week, 6 weeks	1. Muscle tone 2. Walking cadence 3. Walking speed 4. Sensorimotor recovery
Kim et al. (2014)	N: 32 rTMS: 22 CON: 10	rTMS: 11/11 CON: 6/4	rTMS: 67.4 ± 7.8/0.52 ± 0.42 CON: 64.8 ± 11.7/0.49 ± 0.16	First-ever ischemic cerebellar/brain stem stroke	rTMS	Figure-of-eight coil	Sham rTMS	Frequency: 1 Hz Duration: 15 min x 5 sessions, 5 days	1. Walking ability 2. Balance
Rastgoo et al. (2016)	N: 20 rTMS: 10 CON: 10	rTMS: 8/2 CON: 8/2	rTMS: 54.6 ± 11.75/30.2 ± 18.3 CON: 49.7 ± 11/27.4 ± 20.1	First-ever stroke resulted in unilateral hemiparesis	rTMS	Figure-of-eight coil	Sham rTMS	Frequency: 1 Hz Duration: 20 min x 5 sessions, 5 days	1. Muscle spasticity 2. Walking ability 3. Lower limb functions
Wang et al. (2012)	N: 24 rTMS: 12 CON: 12	rTMS: 7/5 CON: 8/4	rTMS: 64.90 ± 12.37/22.08 ± 13.92 CON: 62.98 ± 10.88/12.00 ± 14.76	Unilateral hemiparesis secondary to cerebrovascular accident	rTMS + task-oriented treatment	Figure-of-eight coil	Sham rTMS + task-oriented treatment	Frequency: 1 Hz Duration: 10 min x 10 sessions, 2 weeks	1. Motor performance 2. Walking performance
**2. High-frequency rTMS**									
Chieffo et al. (2014)	N: 10 rTMS: 10 CON: 10	NA	62.2 ± 10.23/21 ± 7.29	First-ever stroke in the middle cerebral artery	rTMS	H-coil	Sham rTMS	Frequency: 20 Hz Duration: 30 min x 10 sessions, 3 weeks	1. Lower limb functions 2. Walking ability
Choi et al. (2016)	N: 30 rTMS: 15 CON: 15	rTMS: 14/1 CON: 13/2	rTMS: 67.1 ± 3.8/49.6 ± 28.3 CON: 68.7 ± 5.2/44.0 ± 29.9	Chronic stroke in in the middle cerebral artery	rTMS	Figure-of-eight coil	Sham rTMS	Frequency: 10 Hz Duration: 10 min x 10 sessions, 2 weeks	1. Balance
Goh et al. (2020)	N: 15	10/5	57.7 ± 9.7/ 22.8 ± 16.7	First-ever left hemispheric stroke	rTMS	Double-cone coil	NA	Frequency: 5 Hz Duration: 16 min x 3 sessions, 7 ± 2days	1. Gait speed
Ji et al. (2016)	N: 30 rTMS: 15 CON: 15	rTMS: 8/7 CON: 9/6	rTMS: 53.80 ± 8.07/1.80 ± 0.77 CON: 56.33 ± 10.98/1.66 ± 0.61	Ischemic and hemispheric stroke	rTMS	Figure-of-eight coil	Sham rTMS	Frequency: 10 Hz Duration: 15 min x 5 sessions per week, 4 weeks	1. Static balance 2. Dynamic balance
Kakuda et al. (2013)	N: 19	10/9	56.2 ± 11.9/61.0 ± 26.1	A single symptomatic supratentorial stroke	rTMS + mobility training	Double-cone coil	NA	Frequency: 10 Hz Duration: 20 min x 20 sessions, 13 days	1. Walking velocity 2. Lower limb functions
Wang et al. (2019)	N: 14 rTMS: 8 CON: 6	rTMS: 7/1 CON: 4/2	rTMS: 53.5 ± 13.7/31.8 ± 24.0 CON: 54.7 ± 12.2/25.3 ± 15.7	Unilateral hemiparesis secondary to stroke	rTMS	Figure-of-eight coil	Sham rTMS	Frequency: 5 Hz Duration: 15 min x 3 sessions per week, 3 weeks	1. Walking speed 2. Gait symmetry 3. Lower limb functions 4. Muscle activity

*N, number; M, male; F, female; rTMS, repetitive transcranial magnetic stimulation; CON, control interventions; NA, not available*.

**Table 3:** The primary outcomes of the included studies.

**Trials**	**Primary outcomes**
**Low-frequency rTMS**	
Elkholy et al. (2014)	rTMS significantly improved all the parameters of the stroke patients when compared to the pre-intervention and control groups.
Kim et al. (2014)	Real rTMS intervention significantly improved the walking ability and balance of the stroke patients when compared to pre-intervention and sham stimulation.
Rastgoo et al. (2016)	Real rTMS intervention significantly improved the muscle spasticity and motor function of the stroke patients when compared to pre-intervention and sham stimulation.
Wang et al. (2012)	Real rTMS + task-oriented treatment significantly improved the motor control and walking ability of the stroke patients when compared to pre-intervention and sham rTMS + task-oriented treatment.
**High-frequency rTMS**	
Chieffo et al. (2014)	Real rTMS intervention significantly improved the lower limb functions of the stroke patients when compared to pre-intervention and sham stimulation.
Choi et al. (2016)	Real rTMS intervention significantly improved the balance function of the stroke patients when compared to pre-intervention and sham stimulation.
Goh et al. (2020)	rTMS significantly improved the dual-task gait speed, but not the single-task gait speed of the stroke patients, when compared to pre-intervention.
Ji et al. (2016)	Real rTMS intervention significantly improved both the static and dynamic balance of the stroke patients when compared to pre-intervention and sham rTMS.
Kakuda et al. (2013)	rTMS + mobility training significantly improved walking velocity and lower limb functions of the stroke patients when compared to pre-intervention.
Wang et al. (2019)	Real rTMS intervention significantly improved the walking speed, gait asymmetry, and motor function of the stroke patients when compared to pre-intervention and sham stimulation.

*rTMS, repetitive transcranial magnetic stimulation*.

The main findings of these included studies were further presented in the following two sections, (1) Effects of low-frequency stimulus; (2) Effects of high-frequency stimulus.

#### Effects of Low-Frequency Stimulus

Four studies that investigated the effects of low-frequency stimulus on lower-limb motor dysfunction of stroke patients were included in this review. Wang et al. (2012) first started trials on these patients. Subjects received rTMS or sham rTMS (1 Hz) followed by task-specific training for 2 weeks in total. The results demonstrated that real rTMS combined with task-oriented treatment significantly improved the motor control and walking ability of the stroke patients when compared to pre-intervention and sham rTMS + task-oriented treatment. Two subsequent studies based on a similar rTMS intervention (rTMS with 1 Hz frequency) also reported some positive results. Elkholy et al. (2014) compared the effects of low-frequency rTMS with conventional physical therapy on muscle tone, walking cadence, walking speed, and sensorimotor recovery of stroke patients. Subjects were required to receive 20-min rTMS 3 sessions per week for 6 weeks. They found that rTMS significantly improved all the parameters of the stroke patients when compared to the pre-intervention and control groups. Kim et al. (2014) also demonstrated that 15-min real rTMS intervention for five consecutive days significantly improved the walking ability and balance of the stroke patients. Finally, Rastgoo et al. (2016) further confirmed the effects of low-frequency rTMS on stroke patients using a similar intervention setting with Kim et al. (2014). They indicated that real rTMS intervention significantly improved the muscle spasticity and motor function of the stroke patients when compared to pre-intervention and sham stimulation.

#### Effects of High-Frequency Stimulus

More than half of the included studies (6 out of 10 trials) investigated the effects of high-frequency rTMS on the lower-limb motor ability of stroke patients. Specifically, rTMS at a frequency of 5 Hz was applied in two studies, rTMS at a frequency of 10 Hz were applied in three studies, and the remaining one study applied rTMS at a frequency of 20 Hz.

Two recent studies focused on the effects of rTMS (5 Hz). Wang et al. (2019) found that 15-min real rTMS intervention at three sessions per week for 3 weeks significantly improved the walking speed, gait asymmetry, and motor function of the stroke patients when compared to pre-intervention and sham stimulation. Goh et al. (2020) further confirmed the effects of rTMS on gait speed after the subjects taking part in a 16-min real rTMS intervention for three sessions during 1 week. In terms of the rTMS at a frequency of 10 Hz, positive effects were found in all the three studies although there are some methodological differences. Kakuda and his colleague first start the trial in 2013. Subjects were required to receive a 20-min rTMS and specific mobility training for 20 sessions during 13 days, and they found that rTMS + mobility training significantly improved walking velocity and lower limb functions of the stroke patients when compared to pre-intervention. In 2016, both Choi et al. (2016) and Ji et al. (2016) investigated the effects of rTMS on the balance of stroke patients using a similar intervention setting, and they demonstrated that real rTMS intervention significantly improved the balance function of the stroke patients when compared to pre-intervention and sham stimulation. In addition, Chieffo et al. (2014) compared the effects of 30-min real rTMS intervention (20 Hz) for 10 sessions during 3 weeks with sham rTMS on lower limb functions and walking ability of stroke patients. The results indicated that the real rTMS intervention significantly improved the lower limb functions of the stroke patients when compared to pre-intervention and sham stimulation.

## Discussion

This study primarily reviewed and summarized evidence from previous research articles investigating the effects of rTMS on lower limb motor ability of stroke patients, with the further aim to find out the appropriate rTMS setting that may contribute to more beneficial results.

According to the eligibility criteria, 10 studies that covered 257 stroke patients were included in this systematic review. Although the risk of bias is relatively high, the results of this review primarily confirmed that rTMS has a positive effect on the lower limb motor ability of stroke patients. To be more specific, both low-frequency rTMS (<5 Hz) and high-frequency rTMS (≥5 Hz) added benefits to the muscle function, walking ability, and balance of stroke patients. In terms of the appropriate setting of rTMS, it was found that 15- to 20-min course of rTMS for 2 to 3 weeks was the most common one. In addition, the results of this review also found that 1 Hz and 10 Hz were the most commonly used low and high frequencies, respectively. Nevertheless, only 10 studies were included, which may weaken the validity of the findings in this review. More rigorous trials in this field are warranted for further verification. Regarding the frequency of rTMS, it is also suggested that future studies should investigate the effects of different frequencies of rTMS on the lower limb motor ability of stroke patients. In addition, there are two included studies investigating the effects of combination intervention (e.g., rTMS combined with task-oriented training or rTMS combined with mobility training) on stroke patients, and they also found some positive results (Wang et al., 2012; Kakuda et al., 2013). However, neither of them was compared with the single rTMS intervention, thus whether a better effect exists is still unclear and is worth studying.

The underlying mechanism by which rTMS can add help to the recovery of lower limb motor ability of stroke patients has been widely speculated. It was found that stroke disrupts the balance of activity in the two brain hemispheres (Wang et al., 2019). The hypothesis of interhemispheric competition holds that the motor cortex of the uninfluenced hemisphere is inhibited, while the motor cortex of the affected hemisphere is exaggerated (Wang et al., 2019). Therefore, the reduction of competition between cerebral hemispheres after stroke is considered to be a potential mechanism for functional improvement after stroke (Nowak et al., 2009). rTMS is one of the non-invasive methods to stimulate nerve cells in the superficial brain zones (Barker, 1991). It has been proven that rTMS through the brain can regulate cortical excitability and cortical restoration, with the influence mainly relying on the different frequencies of the stimulation (Chen and Seitz, 2001). Low-frequency rTMS may initiate a short-term decline in cortical excitability of the affected hemisphere, such as motor evoked potential. The inhibition of cerebellar excitability brought by rTMS with low frequency may further improve the ability to adapt to learning in the process of conventional stroke rehabilitation, thereby better-improving walking ability (Wang et al., 2012; Elkholy et al., 2014; Kim et al., 2014; Rastgoo et al., 2016). On the other hand, rTMS with high frequency enhances the amplitude of motor evoked potential and cortical excitability of the uninfluenced hemisphere, potentially reduced the competition and gradually help to improve the motor ability of stroke patients (Kakuda et al., 2013; Chieffo et al., 2014; Choi et al., 2016; Ji et al., 2016; Wang et al., 2019; Goh et al., 2020).

Some limitations that existed in this review need to be addressed. For example, although the citation snowballing method was applied to identify relevant studies in the reference list of all eligible articles, only three databases were used in this study for searching the relevant published literature, which may accidentally leave out some studies. In addition, the small sample size and relatively high risk of bias of these included studies may potentially weaken the validation of these findings.

## Conclusions

In conclusion, the results of this systematic review primarily confirmed the positive effects of rTMS on the lower limb motor ability (e.g., gait and balance) of stroke patients. It was also found that 15- to 20-min course of rTMS for 2 to 3 weeks was the most common one, and 1 Hz and 10 Hz were the most commonly used low and high frequencies, respectively. These results have significant clinical applications for patients with weakened lower-limb mobility after a stroke. Research related to rTMS either on gait or balance is in a fledging period, more rigorous studies are ought to be focused on this field.

## Data Availability Statement

The original contributions presented in the study are included in the article/supplementary material, further inquiries can be directed to the corresponding author.

## Author Contributions

HF, YS, XC, and PY conceived the presented idea, developed the framework, and wrote the manuscript. IB and YG provided critical feedback and contributed to the final version. All authors were involved in the final direction of the paper, contributed to the final version of the manuscript, and have read and agreed to the published version of the manuscript.

## Conflict of Interest

The authors declare that the research was conducted in the absence of any commercial or financial relationships that could be construed as a potential conflict of interest.

## Publisher's Note

All claims expressed in this article are solely those of the authors and do not necessarily represent those of their affiliated organizations, or those of the publisher, the editors and the reviewers. Any product that may be evaluated in this article, or claim that may be made by its manufacturer, is not guaranteed or endorsed by the publisher.

## References

[B1] Akazawa N. Kazuhiro H. Naomi O. Kimiyuki T. Atsushi H. Hideki M. (2017). Relationships between muscle mass, intramuscular adipose and fibrous tissues of the quadriceps, and gait independence in chronic stroke survivors: a cross-sectional study. Physiotherapy 104, 438–445. 10.1016/j.physio.2017.08.009 29290379

[B2] Attal N. Samar S. A. Ciampi D. A. D. Alaa M. Sophie B. Frédérique J.. (2016). Repetitive transcranial magnetic stimulation and transcranial direct current stimulation in neuropathic pain due to radiculopathy. Pain 157,1224–1231. 10.1097/j.pain.0000000000000510 26845524

[B3] Barker A. T. (1991). An introduction to the basic principles of magnetic nerve stimulation. J. Clin. Neurophysiol. 8, 26–37. 10.1097/00004691-199101000-00005 2019648

[B4] Barroso F. O. Diego T. Francisco M. Alguacil-Diego I. M. Roberto C. Cristina S.. (2017). Combining muscle synergies and biomechanical analysis to assess gait in stroke patients. J. Biomech. 63, 98–103. 10.1016/j.jbiomech.2017.08.006 28882330

[B5] Byun S. Jung T. Kim C. Lee Y. (2011). Effects of the sliding rehabilitation machine on balance and gait in chronic stroke patients – a controlled clinical trial. Clin. Rehabil. 25, 408–415. 10.1177/0269215510385850 21131336

[B6] Cha H. G. Kim M. K. (2017). Effects of strengthening exercise integrated repetitive transcranial magnetic stimulation on motor function recovery in subacute stroke patients: a randomized controlled trial. Technol. Health Care 25, 1–9. 10.3233/THC-171294 28106573

[B7] Chen C-Y. Hong P. W-H. Chen C-L. Chou S. W. Wu C-Y.. (2007). Ground reaction force patterns in stroke patients with various degrees of motor recovery determined by plantar dynamic analysis. Chang. Gung. Med. J. 30, 62–72. 17477031

[B8] Chen R. Seitz R. J. (2001). Changing cortical excitability with low-frequency magnetic stimulation. Neurology 57, 379–380. 10.1212/WNL.57.3.379 11502898

[B9] Chieffo R. De Prezzo S. Houdayer E. Nuara A. Di Maggio G. Coppi E.. (2014). Deep repetitive transcranial magnetic stimulation with H-coil on lower limb motor function in chronic stroke: a pilot study. Arch. Phys. Med. Rehabil. 95, 1141–1147. 10.1016/j.apmr.2014.02.019 24625546

[B10] Choi C. M. Kim J. H. Lee J. K. Lee B. Y. Kee H. S. Jung K. I.. (2016). Effects of repetitive transcranial magnetic stimulation over trunk motor spot on balance function in stroke patients. Ann. Rehabil. Med. 40, 826–834. 10.5535/arm.2016.40.5.826 27847712 PMC5108709

[B11] Clément N. Emmanuelle D. Gregory S. Annick R. Elise L. Sonia D.. (2018). Effects of low- and high-frequency repetitive transcranial magnetic stimulation on long-latency auditory evoked potentials. Neurosci. Lett. 686, 198–204. 10.1016/j.neulet.2018.09.002 30219485

[B12] Elkholy S. H. Atteya A. A. Hassan W. A. Sharaf M. Gohary A. M. E. (2014). Low rate Repetitive Transcranial Magnetic Stimulation (rTMS) and gait rehabilitation after stroke. Int. J. Neurorehabilitation 1:109. 10.4172/2376-0281.10001109

[B13] Goh H. T. Connolly K. Hardy J. McCain K. Walker-Batson D. (2020). Single session of repetitive transcranial magnetic stimulation to left dorsolateral prefrontal cortex increased dual-task gait speed in chronic stroke: a pilot study. Gait Posture 78, 1–5. 10.1016/j.gaitpost.2020.02.020 32146157

[B14] Ji S. G. Shin Y. J. Kim M. K. (2016). The effects of repetitive transcranial magnetic stimulation on balance ability in acute stroke patients. Korean Soc. Phys. Med. 11, 11–17. 10.13066/kspm.2016.11.3.11

[B15] Kakuda W. Abo M. Watanabe S. Momosaki R. Hashimoto G. Nakayama Y.. (2013). High-frequency rTMS applied over bilateral leg motor areas combined with mobility training for gait disturbance after stroke: a preliminary study. Brain Inj. 27, 1080–1086. 10.3109/02699052.2013.794973 23834634

[B16] Kelly-Hayes M. Beiser A. Kase C. S. Scaramucci A. D'Agostino R. B. Wolf P. A. (2003). The influence of gender and age on disability following ischemic stroke: the Framingham study. J. Stroke Cerebrovasc. Dis. 12, 119–126. 10.1016/S1052-3057(03)00042-9 17903915

[B17] Kim C. M. Eng J. J. (2004). Magnitude and pattern of 3D kinematic and kinetic gait profiles in persons with stroke: relationship to walking speed. Gait Posture 20, 140–146. 10.1016/j.gaitpost.2003.07.002 15336283 PMC3167865

[B18] Kim W. S. Jung S. H. Oh M. K. Min Y. S. Lim J. Y. Paik N. J. (2014). Effect of repetitive transcranial magnetic stimulation over the cerebellum on patients with ataxia after posterior circulation stroke: a pilot study. J. Rehabil. Med. 46, 418–423. 10.2340/16501977-1802 24658396

[B19] Machado S. Bittencourt J. Minc D. Cláudio E. P. Riberio P. (2008). Therapeutic applications of repetitive transcranial magnetic stimulation in clinical neurorehabilitation. Funct. Neurol. 23, 113–122. 19152730

[B20] Mansur C. G. Fregni F. Boggio P. S. Riberto M. Gallucci-Neto J. Santos C. M.. (2005). A sham stimulation-controlled trial of rTMS of the unaffected hemisphere in stroke patients. Neurology 64, 1802–1804. 10.1212/01.WNL.0000161839.38079.92 15911819

[B21] Naoki A. Kazuhiro H. Naomi O. Kimiyuki T. Hideki M. Masaki M. (2018). Muscle mass and intramuscular fat of the quadriceps are related to muscle strength in non-ambulatory chronic stroke survivors: a cross-sectional study. PLoS ONE 13:e0201789. 10.1371/journal.pone.0201789 30071100 PMC6072321

[B22] Nowak D. A. Grefkes C. Ameli M. Fink G. R. (2009). Interhemispheric competition after stroke: brain stimulation to enhance recovery of function of the affected hand. Neurorehabil. Neural Repair 23, 641–656. 10.1177/1545968309336661 19531606

[B23] Patterson K. K. Parafianowicz I. Danells C. J. Closson V. Verrier M. C. Staines W. R.. (2008). Gait asymmetry in community-ambulating stroke survivors. Arch. Phys. Med. Rehabil. 89, 304–310. 10.1016/j.apmr.2007.08.142 18226655

[B24] Rastgoo M. Naghdi S. Ansari N. N. Olyaei G. Jalaei S. Forogh B.. (2016). Effects of repetitive transcranial magnetic stimulation on lower extremity spasticity and motor function in stroke patients. Disabil. Rehabil. 38, 1918–1926. 10.3109/09638288.2015.1107780 26878554

[B25] Sasaki N. Abo M. Hara T. Yamada N. Niimi M. Kakuda W. (2016). High-frequency rTMS on leg motor area in the early phase of stroke. Acta Neurol. Belg. 117, 189–194. 10.1007/s13760-016-0687-1 27502413

[B26] Scherbakov N. Haehling S. Anker S. D. Dirnagl U. Doehner W. (2013). Stroke induced sarcopenia: muscle wasting and disability after stroke. Int. J. Cardiol. 170, 89–94. 10.1016/j.ijcard.2013.10.031 24231058

[B27] Song Y. Sun D. István B. Thirupathi A. Liang M. Teo E. C.. (2020). Current evidence on traditional Chinese exercise for cancers: a systematic review of randomized controlled trials. Int. J. Env. Res. Public Health 17:5011. 10.3390/ijerph17145011 32664702 PMC7400020

[B28] Tung Y.-C. Lai C.-H. Liao C.-D. Huang S.-W. Liou T.-H. Chen H.-C. (2019). Repetitive transcranial magnetic stimulation of lower limb motor function in patients with stroke: a systematic review and meta-analysis of randomized controlled trials. Clin. Rehabil. 33, 1102–1112. 10.1177/0269215519835889 30864462

[B29] Von Schroeder H. P. Coutts R. D. Lyden P. D. Billings E. Nickel V. L. (1995). Gait parameters following stroke: a practical assessment. J. Rehabil. Res. Dev. 32, 25–31. 7760264

[B30] Wade D. T. Collen F. M. Robb G. F. Warlow C. P. (1992). Physiotherapy intervention late after stroke and mobility. BMJ Clin. Res. 304, 1179–1180. 10.1136/bmj.304.6835.1179-c 1559090 PMC1881332

[B31] Wang R. Y. Tseng H. Y. Liao K. K. Wang C. J. Lai K. L. Yang Y. R. (2012). rTMS combined with task-oriented training to improve symmetry of interhemispheric corticomotor excitability and gait performance after stroke: a randomized trial. Neurorehabil. Neural Repair 26, 222–230. 10.1177/1545968311423265 21974983

[B32] Wang R. Y. Wang F. Y. Huang S. F. Yang Y. R. (2019). High-frequency repetitive transcranial magnetic stimulation enhanced treadmill training effects on gait performance in individuals with chronic stroke: a double-blinded randomized controlled pilot trial. Gait Posture 68, 382–387. 10.1016/j.gaitpost.2018.12.023 30586670

[B33] Wolfe C. D. (2000). The impact of stroke. Br. Med. Bull. 56, 275–286. 10.1258/0007142001903120 11092079

